# Gene-Based Single Nucleotide Polymorphism Markers for Genetic and Association Mapping in Common Bean

**DOI:** 10.1186/1471-2156-13-48

**Published:** 2012-06-26

**Authors:** Carlos H Galeano, Andrés J Cortés, Andrea C Fernández, Álvaro Soler, Natalia Franco-Herrera, Godwill Makunde, Jos Vanderleyden, Matthew W Blair

**Affiliations:** 1 Centre of Microbial and Plant Genetics, Kasteelpark Arenberg 20, 3001, Heverlee, Belgium; 2 Evolutionary Biology Centre, Uppsala University, SE-751 05, Uppsala, Sweden; 3 Sugarbeet and Bean Research Unit, USDA-ARS East Lansing, MI, 4882, USA; 4 International Center for Tropical Agriculture (CIAT) Bean Project; A.A, 6713, Cali, Colombia; 5 Crop Breeding Institute, P.O.Box CY550, Harare, Zimbabwe; 6 Current address: Department of Plant Breeding, Emerson Hall, Cornell University, Ithaca, NY, USA

## Abstract

**Background:**

In common bean, expressed sequence tags (ESTs) are an underestimated source of gene-based markers such as insertion-deletions (Indels) or single-nucleotide polymorphisms (SNPs). However, due to the nature of these conserved sequences, detection of markers is difficult and portrays low levels of polymorphism. Therefore, development of intron-spanning EST-SNP markers can be a valuable resource for genetic experiments such as genetic mapping and association studies.

**Results:**

In this study, a total of 313 new gene-based markers were developed at target genes. Intronic variation was deeply explored in order to capture more polymorphism. Introns were putatively identified after comparing the common bean ESTs with the soybean genome, and the primers were designed over intron-flanking regions. The intronic regions were evaluated for parental polymorphisms using the single strand conformational polymorphism (SSCP) technique and Sequenom MassARRAY system. A total of 53 new marker loci were placed on an integrated molecular map in the DOR364 × G19833 recombinant inbred line (RIL) population. The new linkage map was used to build a consensus map, merging the linkage maps of the BAT93 × JALO EEP558 and DOR364 × BAT477 populations. A total of 1,060 markers were mapped, with a total map length of 2,041 cM across 11 linkage groups. As a second application of the generated resource, a diversity panel with 93 genotypes was evaluated with 173 SNP markers using the MassARRAY-platform and KASPar technology. These results were coupled with previous SSR evaluations and drought tolerance assays carried out on the same individuals. This agglomerative dataset was examined, in order to discover marker-trait associations, using general linear model (GLM) and mixed linear model (MLM). Some significant associations with yield components were identified, and were consistent with previous findings.

**Conclusions:**

In short, this study illustrates the power of intron-based markers for linkage and association mapping in common bean. The utility of these markers is discussed in relation with the usefulness of microsatellites, the molecular markers by excellence in this crop.

## Background

Single nucleotide polymorphisms (SNPs) are the most abundant class of polymorphic sites in any genome. They have become a powerful tool in genetic mapping, association studies, diversity analysis and positional cloning [1]. SNPs are usually biallelic, therefore less polymorphic than SSRs. However, this limitation is compensated by the ability to use more markers and to build SNP haplotypes [2]. The discovery of SNPs in candidate genes or transcript sequences (ESTs) has been a recurrent strategy in plant genetics mainly because gene-based SNP markers could themselves be causative SNPs for traits. In legumes, gene-based markers have been used to develop transcript maps in chickpea (*Cicer arietinum* L.) [3] and soybean (*Glycine max* L.) [4]. QTL analysis in cowpea (*Vigna unguiculata* L.) [5], association mapping in *Medicago truncatula*[6] and synteny analysis in common bean (*Phaseolus vulgaris* L.) [7,8] have been reported as well.

In common bean, EST libraries of the Mesoamerican genotype Negro Jamapa 81 and the Andean genotype G19833 were used to establish the first consolidated resource of SNP markers [9]. Some of these SNPs were mapped in the population DOR364 × G19833 using mismatch cleavage nuclease CEL I [10] and single strand conformational polymorphism (SSCP) [7]. Other approaches to identify SNPs using CAPS, dCAPS, or size polymorphism, were developed comparing EST libraries from different legumes [11] and selecting the ESTs that presented homology with genes from *Arabidopsis thaliana* and maize (*Zea mays* L.) [8]. Later, Hyten et al. [12] reported a high-throughput SNP discovery platform using a reduced representation library from multiple rounds of nested digestions with sequencing carried out by 454 pyrosequencing and Solexa technologies. More recently, Cortés et al. [13] reported a diversity analysis using a competitive allele specific PCR (KASPar) to evaluate 94 SNPs derived from ESTs and drought genes. However, low polymorphism has constrained the utility of these markers. Nevertheless, this constraint can be avoided by means of a deeper exploration of the intronic regions.

A medium-throughput technique for testing candidate genes with modest multiplexing and minimal assay setup costs is the Sequenom MassARRAY system [14]. In this approach, a region is amplified and then a single-base primer extension is performed using modified deoxyribonucleoside triphosphates that increase the discriminating resolution by means of a mass spectrometer. The Sequenom platform has been used for SNP validation in sugarcane (*Saccharum officinarum* L.) [15], diversity studies in castor bean (*Ricinus communis*) [16] and marker assisted selection in soybean [17]. In common bean, the Sequenom platform has recently been used to evaluate 132 SNPs for association with common bacterial blight resistance [18].

Because of marker abundance, one of the most common applications of SNPs is association mapping (AM). In this approach, the correlation between markers, genes and traits is statistically accessed in unrelated genotypes. Ancestral recombination and natural genetic diversity within populations constitute the basis for the identification of non-random co-segregation of alleles between loci and traits [19]. The extent of this non-random association, also known as linkage disequilibrium (LD), depends on the mating system, the mutation, recombination, and migration rates, the patterns of selection and the degree of population structure [20]. For instance, the natural decay of LD with physical distance occurs in inbreeding species at a considerably slower rate than in outbreeding species because the effective recombination rate in inbreeding species is severely reduced. This means that within few generations a self-fertilizing population is expected to be a collection of homozygous lines [21]. Therefore, much of the theory and practice of AM has been established in heterozygous outbreeding species such as maize [22,23]. Efforts to apply AM in inbreeding species have been relatively restricted. Some outstanding cases are found in *Arabidopsis*[24], barley (*Hordeum vulgare* L.) [25] and rice (*Oryza sativa* L.) [26]. Nevertheless, a thorough and well-designed exploration of AM is missing in common bean, especially with gene-based SNP markers.

The objectives of this study were to: 1) develop a set of intron-based SNP markers at target genes in common bean; 2) map these genes in the core linkage map DOR364 × G19833 and in the consensus map; 3) evaluate the utility of the corresponding intron-based SNP markers in relation with SSRs; and 4) explore the feasibility of the AM approach using the gene-based SNP markers in a self-fertilizing, non-model crop.

## Results

### Gene-based marker evaluation

A total of 313 pairs of primers were designed flanking the intronic regions of 271 common bean target genes. Introns were putatively identified based on the soybean genome (Additional file 1). In addition, 55 pairs of primers were designed over 33 genes involved in the nodulation process in model legumes [27,28]; 63 pairs of primers were designed over 48 transcription factors identified under phosphorus stress [29]; and 195 pairs of primers were designed over 190 putative soybean genes involved in nodule development [30]. Pilot amplification on these 313 intronic regions using the control genotypes DOR364, BAT477 and G19833 was successful in 77% of the cases. The 23% failure may be due to the presence of larger introns. The average size of the amplicon was 700 bp, ranging from 140 bp (BSn1) to 2000 bp (BSn311). The amplicons were evaluated on SSCPs and 8.3% were polymorphic for the parents of the inter-gene-pool population DOR364 × G19833. A set of 65 of these regions were sequenced and aligned in the control genotypes. In most cases the intron region was detected and a total of 178 SNPs were found. Allele specific primers were designed in the flanking regions of these SNPs to be used on the Sequenom platform (Additional file 2).

### Linkage mapping

**Figure 1:**
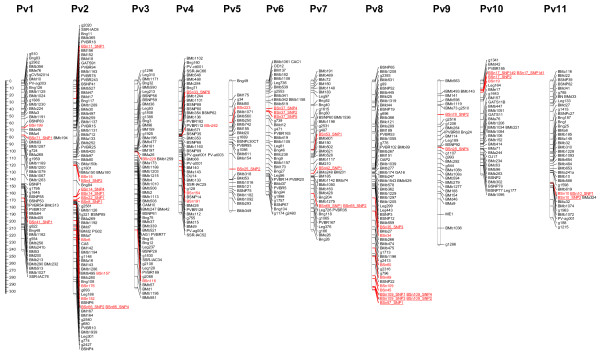
**Linkage map of the DOR364 × G19833 population.** A total of 53 new gene based markers were placed in the linkage map, including 19 markers by SSCP (red) and 34 markers evaluated by Sequenom (red and underlined).

The polymorphic markers were evaluated in the DOR364 × G19833 mapping population using SSCP and Sequenom techniques. A total of 53 new intron-based markers (19 markers identified by SSCP and 34 markers based on the Sequenom technique) were successfully placed in the base linkage map that was previously developed [31] (Figure 1). As expected, the SNPs within the same gene mapped together (i.e. SNPs in the locus BSn37 on Pv6 and Bsn109 on Pv8). The new gene-based markers were well distributed in the genome, with an average of 5 markers per linkage group, ranging from two markers on Pv3 and Pv7, to 13 markers on the Pv2 and Pv8. The final genetic map had 534 marker loci with a full map length of 2,400 cM. Linkage group sizes ranged from 133 cM (Pv10) to 300 cM (Pv8) with an average of 120 cM per linkage group. The number of marker loci per linkage group ranged from 27 on Pv5 to 83 on Pv2. Finally, this new linkage map version of the population DOR364 × G19833 was merged with the previously existing linkage maps of the populations DOR364 × BAT477 and BAT93 × JALO EEP 558 to produce a new consensus map of 1060 markers, thereby increasing the total number of functional markers previously reported by Galeano et al. [31] (Additional file 3).

### Diversity analysis

**Figure 2:**
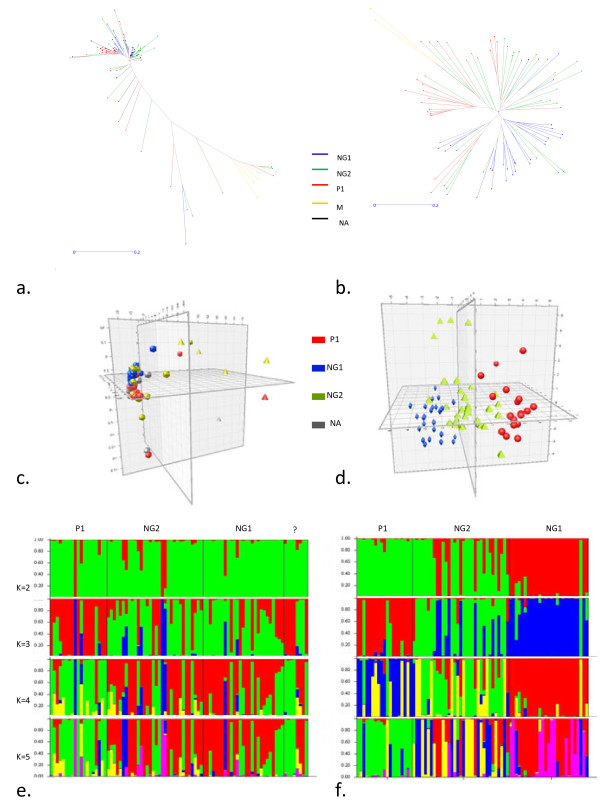
**Genetic Diversity and population structure of the diversity panel. a and b Neighbor-joining trees for the results of 149 SNP and 36 SSR, repectively.** The branches were colored by races: NG Nueva Granada, P1 Peru, and M Mesoamerica. **c** and **d** Principal component analysis for SNP and SSR, respectively. **e** and **f** structure analysis from K = 2 to K = 5 for SNP and SSR, repectively.

In order to evaluate the utility of the corresponding intron-based SNP markers in relation with SSRs and to develop the basis for the AM approach, a diversity analysis was carried out. The diversity panel was mainly formed by Andean genotypes; six Mesoamerican genotypes were included as an out-group to verify the efficiency of the markers to differentiate between gene pools (Additional file 4). A total of 173 new intron-based SNPs were evaluated in the diversity panel using the Sequenom platform. Of these, 22 were monomorphic and six presented a minor allele frequency lower than 0.05. The remaining SNPs had an average polymorphism information content (PIC) of 0.23. Some 17 SNPs presented PIC value less than 0.2 and 18 SNPs had PIC value higher than 0.4. The same genotypes were previously evaluated with 37 SSRs by Blair et al. [32]. In this case, two SSRs were monomorphic, the average number of alleles and PIC were 8.3 and 0.4, respectively. The diversity indexes for the evaluations carried out with SSRs and SNPs are summarized in the Table 1. The fixation index (Fst) between populations was 0.38 and 0.54 for SSRs and SNPs, respectively. The phenetic analysis based on the dissimilarity matrix showed that SSRs provided more resolution, and therefore dispersion, between the accessions (Figure 2a,b). In both cases, the Mesoamerican genotypes could be clearly distinguished from the pool of the Andean genotypes. Consequently, in order to avoid strong population structure effects, the subsequent analyses were constrained to the Andean genotypes.

**Table 1:** Diversity index of SNP and SSR in the Andean diversity panel

**SNP**									
		**N**	**Na**	**Ne**	**I**	**Ho**	**He**	**UHe**	**F**
	**Mean**	81.664	1.993	1.251	0.296	0.085	0.172	0.173	0.656
	**SE**	0.350	0.007	0.022	0.014	0.017	0.011	0.011	0.038
**SSR**									
	**Mean**	75.000	7.865	3.877	1.079	0.018	0.445	0.448	0.904
	**SE**	0.958	1.296	0.684	0.165	0.008	0.060	0.060	0.042

Na = No. of Different Alleles, Ne = No. of Effective Alleles, I = Shannon's Information Index, Ho = Observed Heterozygosity, He = Expected Heterozygosity, UHe = Unbiased Expected Heterozygosity, F = Fixation Index.

Principal component analysis revealed two and three distinguished groups for the SNP and SSR datasets, respectively (Figure 2c,d). The division between the three groups with SSRs was more than for the groups based on the SNP dataset, where one of the groups could possibly correspond to outliers. Similar results were obtained with a Bayesian approach implemented in Structure (Figure 2e,f). In this case, SNPs did not reveal substructure within our Andean panel, but SSRs perfectly matched the expectations for the three different Andean races. The most plausible number of populations was calculated using the method of Evanno et al. [33], and confirmed the previous observation (Additional file 5). In short, the genetic variation that is captured by the SSRs is mostly driven by the race structure of the gene pool. This is not the case for the SNP markers.

A subsequent linkage disequilibrium analysis and association study only included the 110 SNPs and 24 SSRs that had a minor allele frequency higher than 0.05. The mean *r*^2^ value between SNPs and SSRs was 0.18 and 0.025, respectively. Linkage disequilibrium was lower between SSRs than between SNPs, mainly because of the number of markers that were considered. Finally, haplotype blocks were clearly identified based on the linkage disequilibrium between neighbor SNPs (Additional file 6). For this purpose, SNPs were arranged according to loci and the recombination distance (cM) between them. The SNP markers revealed extended linkage disequilibrium within linkage groups Pv2 and Pv4, and between linkage groups Pv1 and Pv2, and Pv2 and Pv8.

### Association analysis

GLM generally presented lower *p* values than MLM. Additionally, GLM revealed more than 100 associations with significance above 95% (data not shown). Therefore a Bonferroni correction was done to reduce the number of false positives. According to the GLM, 16 loci presented 53 associations with the evaluated traits. A total of 30 associations were identified across both environments, and 8 and 15 associations were unique for the irrigation and drought treatments, respectively. The markers specifically associated with one of the conditions are being evaluated in relation with other physiological traits and in different drought conditions (G. Makunde, *et al.* in preparation). On the other hand, this study has focused on markers that presented associations in both environments to minimize the environmental effects on the associations. Interestingly, 10 of the 12 loci that presented associations in both environments were SSRs. The other two were genes. Some markers presented associations with more than one trait. For instance, the marker BM143 at Pv2 was associated with DF, DM, EP, PP and SPL; and the marker BM160 at Pv7 was associated with DM, EP, PP, SP and SPL (Additional file 7).

On the other hand, according to the MLM, 28 loci showed 66 associations. Of them, 28 were found in both environments and 22 and 16 associations were only significant for irrigation and drought, respectively (Additional file 8). A total of 10 loci presented associations in both environments, and contrary to the GLM results, seven were target genes and three were SSRs (Table 2). Some markers presented associations with more than one trait, as well. Specifically, BSn66_SNP2 at Pv2 was associated with DM, EP, PP, SP, SPL and yield; and BSn44_2 at Pv3 was associated with DF, DM, P100, PLA, SP, SPL and yield. Additionally, comparing the results of GLM and MLM models, two markers BM143 and BSn244_2, presented significant associations in both analyses. For the remaining comparisons, GLM was omitted because it does not consider co-ancestry as a co-factor, and therefore the rate of false-positives is inflated when using this method.

**Table 2:** Association analysis based on MLM

**Trait**	**Marker**	**LG**	**Environment**	**R2**	**p value**		**Putative function**
DF	BM143^a^	2	drought	0.4643	0.0315	*	-
DF	BM143^a^	2	irrigation	0.4747	0.0302	*	-
DF	BSn109_SNP4	8	drought	0.2035	0.0023	**	-
DF	BSn109_SNP4	8	irrigation	0.2016	0.0024	**	-
DF	BSn244_2^a^	3	drought	0.1383	0.0015	**	acyl acp-thioesterase
DF	BSn244_2^a^	3	irrigation	0.1457	0.0011	**	acyl acp-thioesterase
DM	BSn66_SNP2	2	drought	0.1883	0.0039	**	auxin response factor 2
DM	BSn66_SNP2	2	irrigation	0.2420	0.0010	***	auxin response factor 2
DM	BSn85_SNP2	8	drought	0.1017	0.0239	*	transcription factor bhlh96-like
DM	BSn85_SNP2	8	irrigation	0.1073	0.0218	*	transcription factor bhlh96-like
EP	BSNPK18	8	drought	0.1204	0.0337	*	oxygen-evolving enhancer protein chloroplastic-like
EP	BSNPK18	8	irrigation	0.1235	0.0323	*	oxygen-evolving enhancer protein chloroplastic-like
PLA	BSn244_2	3	drought	0.0527	0.0461	*	acyl acp-thioesterase
PLA	BSn244_2	3	irrigation	0.0694	0.0235	*	acyl acp-thioesterase
PP	BSn14_SNP3	9	drought	0.0918	0.0310	*	-
PP	BSn14_SNP3	9	irrigation	0.0839	0.0484	*	-
PP	BSn66_SNP2	2	drought	0.1552	0.0094	**	auxin response factor 2
PP	BSn66_SNP2	2	irrigation	0.1524	0.0133	*	auxin response factor 2
SP	BSNPK18	8	drought	0.1385	0.0206	*	oxygen-evolving enhancer protein chloroplastic-like
SP	BSNPK18	8	irrigation	0.1114	0.0460	*	oxygen-evolving enhancer protein chloroplastic-like
SPL	BSn244_2	3	drought	0.0568	0.0390	*	acyl acp-thioesterase
SPL	BSn244_2	3	irrigation	0.0667	0.0283	*	acyl acp-thioesterase
SPL	BSn66_SNP2	2	drought	0.1359	0.0192	*	auxin response factor 2
SPL	BSn66_SNP2	2	irrigation	0.1173	0.0391	*	auxin response factor 2
Yield	BSn66_SNP2	2	drought	0.1205	0.0352	*	auxin response factor 2
Yield	BSn66_SNP2	2	irrigation	0.1157	0.0406	*	auxin response factor 2
Yield	BSn85_SNP2	8	drought	0.1050	0.0235	*	transcription factor bhlh96-like
Yield	BSn85_SNP2	8	irrigation	0.0879	0.0421	*	transcription factor bhlh96-like

Trait abbreviations: Days to flowering (DF), days to maturity (DM), pods per plant (PP), seed per pod (SP), seed per plant (SPL), empty pod% (EP), pod length average (PLA)

^a^ Significant association with Bonferroni correction based on GLM.

Genes that were associated with some of the previous traits were submitted to a Blastx search. Four putative proteins were of particular interest. Acyl acp-thioesterase is associated with DF, PLA and SPL, auxin response factor 2 is associated with DM, PP, SPL and yield, transcription factor bhlh96-like is associated with DM and yield, and oxygen-evolving enhancer protein chloroplastic-like is associated with EP and SP.

## Discussion

In this study we reported on a set of 313 intron-flanking gene based markers, specifically based on genes mainly involved in the nodulation pathway in legumes. These markers were evaluated using SSCPs and an allele specific high throughput Sequenom platform. This means that the marker assisted selection community now has two different technologies to further exploit our new resource of molecular markers available. Similar intron-flanking markers have been designed for comparative genomics in other legumes, based on conserved orthologous sequences (COS) [11,34]. In grasses, intron-flanking markers have been evaluated in relation with inter-species diversity and candidate genes within QTLs [35,36].

In terms of linkage analysis, 17% of the SNP markers were placed in the inter gene pool population DOR364 × G19833. In order to identify the putative position of the other SNPs, the linkage map was merged on a consensus map following the methodology reported by Galeano et al. [31]. The synteny analysis allowed *in silico* mapping of the rest of the markers. The consensus map traditionally presents high degree of co-linearity and synteny, and therefore it has become a popular alternative for *in silico* mapping and for association studies in other species, like *Eucalyptus*[37] and wheat (*Triticum* spp.) [38].

The diversity analysis using intron-based SNPs revealed different patterns of diversity compared with the ones described by Blair et al. [32] using SSRs. This may be a consequence of the dissimilar mutation processes that are associated with each type of marker [39]. Therefore, according to Laval et al. [40], (*k*-1) times more biallelic markers are needed to achieve the same genetic distance accuracy as a set of SSR with *k* alleles. In our case, the average number of alleles per SSR locus was about 10. Therefore, we would require [(10–1) * 37] = 333 SNP markers to achieve the same accuracy. In addition, the polymorphism within the intron-based markers could be constrained more extensively than the polymorphism within non-genic regions. Similar results were reported by Cortes et al. [13], where the SNPs were able to differentiate between the Mesoamerican and the Andean gene pools, but the SSRs were more powerful for the identification of races within gene pools. Therefore, it was proposed to use SNP markers at the inter-gene pool level and SSR markers at the intra-gene pool scale in order to explore the diversification and domestication history of the species. In maize, Hamblin et al. [41] reported that SSRs performed better at clustering germplasm and provided more resolution than SNPs, something that has been observed in this study for the case of common bean, as well. Additionally, Jones et al. [42] compared SSRs and SNPs in maize and showed that SNPs can provide more high-quality markers. They suggested that the relative loss in polymorphism compared with SSRs may be compensated by increasing the numbers of SNPs and using SNP haplotypes. Our combination of multiple markers from the same gene and from different genes allowed us to detect the corresponding haplotype blocks, and therefore support this thesis. In short, our results are in line with previous evidence that supports the hypothesis according to which SNPs and SSRs are complementary, non-mutually exclusive, markers that must be chosen based on the ultimate practical purpose. In this sense, we emphasize that the use of one or the other marker does not only depend on the level at which the comparisons will be made, but also on the nature of the comparisons.

Population structure analysis is a key factor for association analysis in plants, in order to minimize type I and II errors between candidate molecular markers and traits of interest [19]. In common bean, the diversification across the Americas and the independent domestication of the wild relatives in two distinct centers gave origin to two main gene pools, the Andean and the Mesoamerican, with extensive race sub-division. Several studies have reported that the Andean beans are more diverse than the Mesoamerican ones [13,32].

Similar trends are theoretically expected in terms of linkage disequilibrium. In the current study, the level of LD in the Andean panel was slightly higher than what previous analyses revealed using AFLP screenings of wild and domesticated accessions [43]. This difference is mainly due to the type of markers and the sample size that were used in each case. Rossi et al. [43] additionally reported higher levels of LD in the Andean gene pool, compared with the Mesoamerican, suggesting that the former originated prior domestication. Analogous correlations between population sub-division and LD decay have been found between tropical and temperate germplasm in maize [44], among *O. sativa ssp. indica* and *O. sativa ssp. japonica*[45], and between two-row and six-row barley [46]. In short, the Andean gene pool offers *per se* an interesting spectrum to look for adaptive variation, at the same time that the confusing effect of sub-structure is minimized.

A recurring issue with the use of QTL data is that different parental combinations or/and experiments conducted in distinct environments often result in the identification of partly or wholly non-overlapping sets of QTLs [47]. Therefore, it is important to explore constitutive QTLs across different environments. In this sense, our field trials offered us the possibility to identify constitutive marker-trait associations because correlations were contrasted across two environments, drought and irrigation. This sort of designs is particularly useful for marker assisted selection (MAS), as was demonstrated in rice [48].

In terms of association mapping models, we used two approaches: GLM and MLM. The GLM presented more significant *p* values and therefore more associations. However, after Bonferroni correction just two markers were detected in common with the MLM results. This finding is in accordance with the results of previous studies [49,50] and indicates that the GLM approach is inappropriate for association mapping in the examined plant species, because the resulting proportion of spurious marker-phenotype associations is considerably higher than the nominal type I error rate. The MLM used here, using as co-factors the kinship matrix (K) and STRUCTURE (Q), revealed interesting results. However, recent studies reported that new models combining K and the 10 principal components (Q_10_) were the best approaches to control the rate of false positives [51,52]. Additionally, although we found some significant association based on high *p* value using MLM, multiple testing needs to be used to control the genome-wide type I error rate (GWER) [53].

Interestingly, the markers BSn66_SNP2 and BM143 were near previous QTL analyses for days to flowering and days to maturity, in different bi-parental populations nearby or flanking the same loci in the same linkage group [54-57]. Additionally, QTLs for yield components such as seed weight and seed per pod have also been reported close to these loci [55,58,59]. In terms of functional genomics, the locus BSn66 is an auxin response factor 2 (ARF2), one member of the family of transcription factors that bind to auxin responsive elements (AuxREs) in the promoter sequences of auxin regulated genes [60]. The ARF gene family has been repeatedly associated with flower and fruit maturation and development [61-63]. For instance, the *arf2* mutants presented enlarged rosette leaves, thickened inflorescence stems, delayed flowering and senescence, reduced fertility and increased seed size [64,65].

In a similar way, SNP marker BSn85_SNP2 on Pv8 is near QTLs for days to maturity and in addition seed weight has been reported nearby this locus [55,56]. The locus BSn85 putatively codifies a basic helix-loop-helix (bHLH) transcription factor. Members of the bHLH gene family are particularly relevant because they interact with the light-activated phytochrome, and therefore control various facets of the photomorphogenic response, including seed germination, seedling deetiolation, shade avoidance and photoperiodic control of plant growth [66,67]. Recently, the interaction of ARF with bHLH transcription factors has been reported in the context of plant growth [62]. These examples of functional congruence and co-localization of some of the associated loci with formerly identified QTLs validate our approach. Even more interesting is the fact that association studies in common bean, specifically within the Andean gene pool, are an excellent alternative to find QTLs based on candidate genes. Pioneer association results in common bean were obtained for SNP markers associated with common bacterial blight (CBB) resistance [18].

Although the sampling in our study was not exhaustive, similar successful studies with small sample sizes have been reported extensively. For example, several SNP markers were associated with oleic acid using 94 genotypes of peanut (*Arachis hypogaea*) from 4 botanical varieties [68], and makers associated with malting quality where found in barley using germplasm sets of 85 genotypes on average [69]. The main advantage of the small, carefully chosen, association mapping panels is the efficacy and affordability with which plant germplasm is used. In some other cases, like in barley, more individuals (approximately 300 lines) are desired [46,70]. However, the final choice of the size of the population depends on the relatedness of the individuals, the extent of linkage disequilibrium, the type of study, and the polymorphism of the markers. We have demonstrated that because of its self-crossing nature, common bean is not really demanding in this aspect, and allows working with medium size populations.

Additionally, considering the population size and low genome coverage, the parental information of the lines will improve the accuracy of the results. This approach has been used particularly in livestock species, with models that integrate data on phenotypes, genotypes and pedigree information. Such information can be combined with genomic data for greater detection power and estimation precision through a properly scaled and augmented relationship matrix [71]. Therefore, this parental information will be very important for association and genome selection approaches in common bean. Unfortunately, at this stage parental information was not available for the materials considered in the present study because they were landrace genotypes collected from farmers and market places.

## Conclusions

In brief, our results indicated that intron-flanking markers are a useful tool for linkage mapping, genetic diversity and association analysis. As the number of genomic sequence resources dramatically increase for major and minor crop species, a larger number of intronic and inter-genic markers will become available to plant geneticists and breeders. Here we have offered a pipeline to mine this resource. Ultimately, this initiative will contribute to close the gap between structural polymorphism and functional diversity.

## Methods

### Plant material

A diversity panel consisting of 93 genotypes was evaluated in this study, mainly consisting of 80 Andean genotypes previously characterized using SSRs [32] and 13 parental lines commonly used in breeding programs at the International Center for Tropical Agriculture (CIAT) (Additional file 4). DNA extraction involved the germination of ten seeds randomly selected from each accession, and pre-germinated on germination paper under dark conditions. The first trifoliate leaves of 8-day-old seedlings were collected and grounded in liquid nitrogen for DNA extraction. DNA was extracted and re-suspended in TE buffer as reported by Galeano et al. [10]. The quality was evaluated on 0.8% agarose gel and quantified by Hoescht H 33258 dye on a Hoefer DyNA fluorometer (DNA Quant™ 200. San Diego, CA). DNA was diluted to 10 ng/μl for further procedures.

### Gene based markers

Four different classes of genes were used. One consisted of genes from the nodulation pathway and involved in Nod factor perception, signal transduction and calcium signal interpretation as reported in legumes by Stacey et al. [27] and Kouchi et al. [28]. Another class corresponded to a sub-set of 372 root transcription factors (TF) reported in common bean by Hernandez et al. [29]. In addition, a set of 162 soybean putative regulatory genes, involved in root hair cell infection, was included [30]. A set of 179 nodule-specific expressed sequences from the common bean, listed in PhvGI Library Expression, were also included (http://compbio.dfci.harvard.edu/cgi-bin/tgi/libtc.pl?db=phvest). All these sequences were downloaded from the NCBI database and compared with the common bean EST assembly [7]. The selected common bean sequences were aligned with the corresponding genome region in soybean (http://www.phytozome.net/soybean) to identify the putative location of exons and introns using Geneious software [72]. A total of 313 exons-anchored primers were designed in order to amplify the intronic regions and named with the prefix Bsn (Additional file 2).

### Genotyping

The gene based markers listed above were evaluated in the genotypes DOR364, BAT477, and G19833. The PCR conditions, agarose gel electrophoresis and SSCP technique were carried out as reported by Galeano et al. [7]. The PCR amplicons were sequenced using BigDye Terminator chemistry with AmpliTaq-FS DNA polymerase (Applied Biosystems) and resolved on an Applied Biosystems Automated 3730 DNA Analyzer at the Cornell University Biotechnology Resource Center. The sequences were aligned and SNPs were detected.

The 93 genotypes (diversity panel) were evaluated for SNPs using the MassARRAY platform of Sequenom (San Diego, USA) at the VIB Vesalius Research Center, Leuven, Belgium. Sequences of minimum 50 bp up and downstream from the SNP were used for primer design using Sequenom MassARRAY Assay Design 3.1 software with default parameters. The markers were named as mentioned above, plus an indication of the SNP within the amplicon (i.e. Bsn4_SNP1). The primer information for Sequenom genotyping is provided in Additional file 3. The genotyping was performed according to the iPLEX protocol from Sequenom (available at http://www.sequenom.com/) in the diversity panel of 93 genotypes. Quality control criteria were adopted using water as negative control and inter-plate duplicates. Additionally, 24 SNPs designed by Cortés et al. [13] were evaluated in the same diversity panel using KASPar technology (markers BSNK).

### Linkage analysis

The SNPs detected between the genotypes DOR364 and G19833 were evaluated in the corresponding mapping population using the SSCP and MassARRAY methodologies described above. The segregation data was used to place the new markers on the established genetic map for the DOR364 × G19833 population (87 RILs) reported by Galeano et al. [31]. The linkage analysis and the consensus map were done following the methodology reported by Galeano et al. [31]. The putative position of markers evaluated in the diversity panel that were not placed in the linkage map, was inferred by *in silico* mapping using the synteny analysis reported by Galeano et al. [7,31]. Briefly, the common bean sequences were aligned against the chromosome based assembly of soybean using local blastn, and based on the closest mapped markers, the genetic distance was inferred.

### Genetic diversity and association analysis

The SNPs data generated in the diversity panel were used to estimate population genetics parameters and Hardy Weinberg equilibrium (HWE) using software GenAlEx [73]. Minor allele frequency, allele number, gene diversity, heterozygosity and PIC parameters were determined with PowerMarker 2.25 [74]. Population structure analysis was conducted with STRUCTURE 2.3.2 [75] as described in Cortés et al. [13]. In addition, Evanno test was carried out in order to estimate the optimal K for the structure analysis [33]. A similar analytical pipeline was performed with the genotypic data from 37 microsatellite markers previously evaluated in the diversity panel by Blair et al. [32]. Diversity parameters were compared between both datasets in order to assess how well each type of marker recovered the genetic signals. Finally, linkage disequilibrium standard statistics were calculated for the SNP dataset using the software TASSEL version 3.0 [76].

On the other hand, phenotypic data of 80 Andean genotypes from the core collection was considered (G. Makunde, unpublished data). The trials were carried out at the International Center for Tropical Agriculture (CIAT) in Palmira, Valle de Cauca, Colombia. The experimental design consisted of 9 × 9 lattice with three repetitions each and two environments (drought and irrigated) evaluated in 2009 following the same methodology reported by Blair et al. [54]. The traits evaluated were days to flowering (DF), days to maturity (DM), pods per plant (PP), seed per pod (SP), seed per plant (SPL), empty pod% (EP), average pod length (PLA), 100 seeds weight (P100), and grain yield. Kinship matrix was calculated as the proportion of allele shared between each pair of lines. Both, general linear model (GLM) and mixed linear model (MLM) were used in the association analysis. In the GLM, the Q matrix was integrated as a co-variable to correct for the effects of population substructure while both Q and K matrices were used in the MLM to correct for both population and family structure. These analyses were carried out with TASSEL and Bonferroni corrections were done to account for multiple comparisons. Finally, the putative functions and ontology of the significantly associated genes were evaluated with Blas2GO software version 2.5.0 [77]. The sequence alignments and editions were done with Genious software version 5.5.6.

## Competing interest

The authors declare that they have not competing interest.

## Authors’ contribution

CHG, MWB and JV designed the study. CHG, NF, GM performed the experiments. CHG, AJC, ACF and AS analyzed the data and contributed to data interpretation and discussion. CHG, AJC, GM, MWB and JV wrote the manuscript. All authors approved the final version of manuscript.

## Supplementary Material

Additional file 1 313 intron-flaking primers. Click here for file

Additional file 2 Sequenom primers list. Click here for file

Additional file 3 Consensus map. Click here for file

Additional file 4 List of the diversity panel genotypes. Click here for file

Additional file 5 Evanno test for the structure analysis. Click here for file

Additional file 6 **Linkage disequilibrium heat maps(r**^**2**^**vs.p-value).** Click here for file

Additional file 7 GLM results. Click here for file

Additional file 8 MLM results. Click here for file
