# Supplementary figures and images for: Gene-Based Single Nucleotide Polymorphism Markers for Genetic and Association Mapping in Common Bean
Source: BMC Genet. 2012 Jun 26;13:48. doi: 10.1186/1471-2156-13-48 (PMC3464600; doi:10.1186/1471-2156-13-48)

## Slide 1
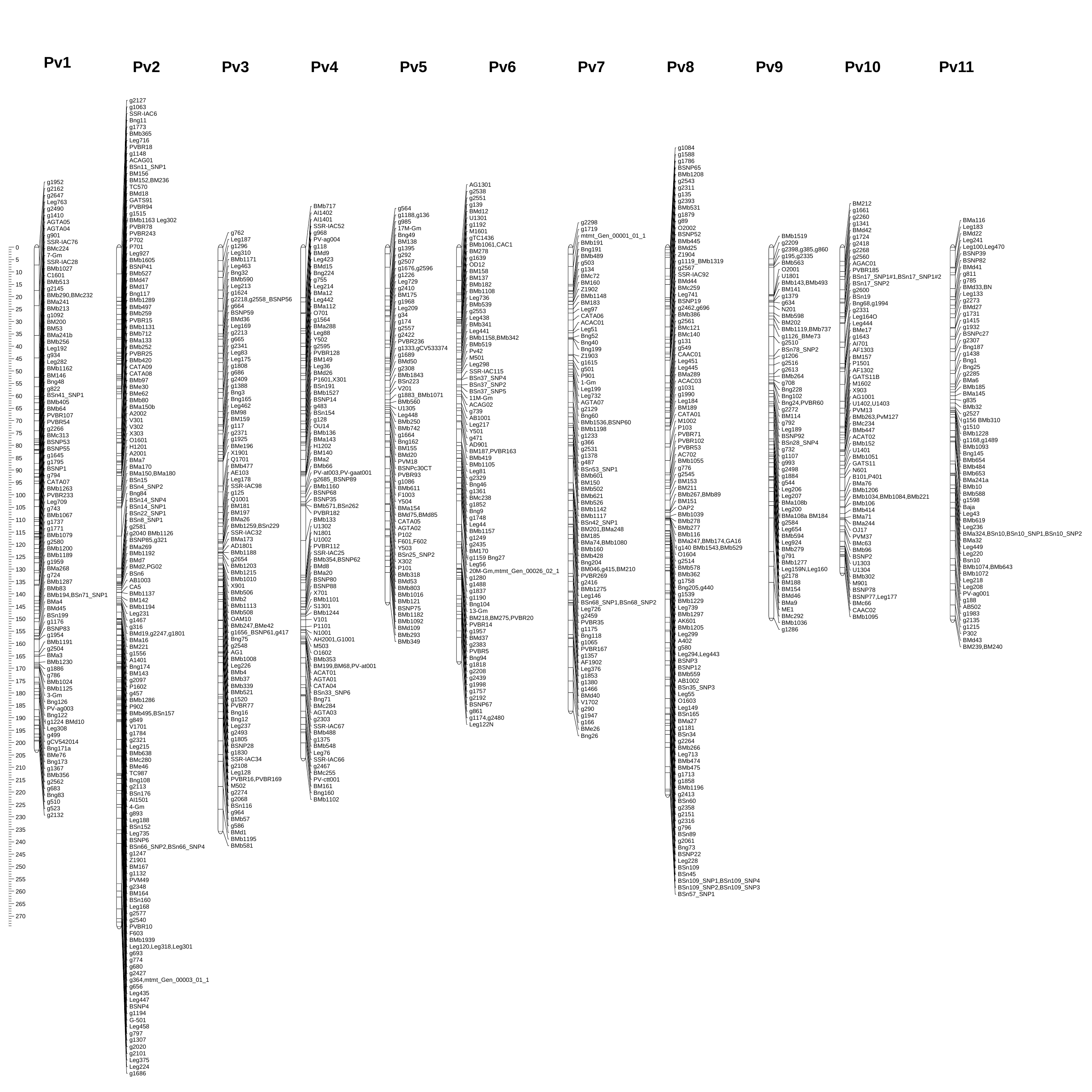

Pv1
Pv2
Pv3
Pv4
Pv5
Pv6
Pv7
Pv8
Pv9
Pv10
Pv11

Supplement: Additional file 3 — Consensus map. [file 1471-2156-13-48-S3.pptx]

## Slide 1
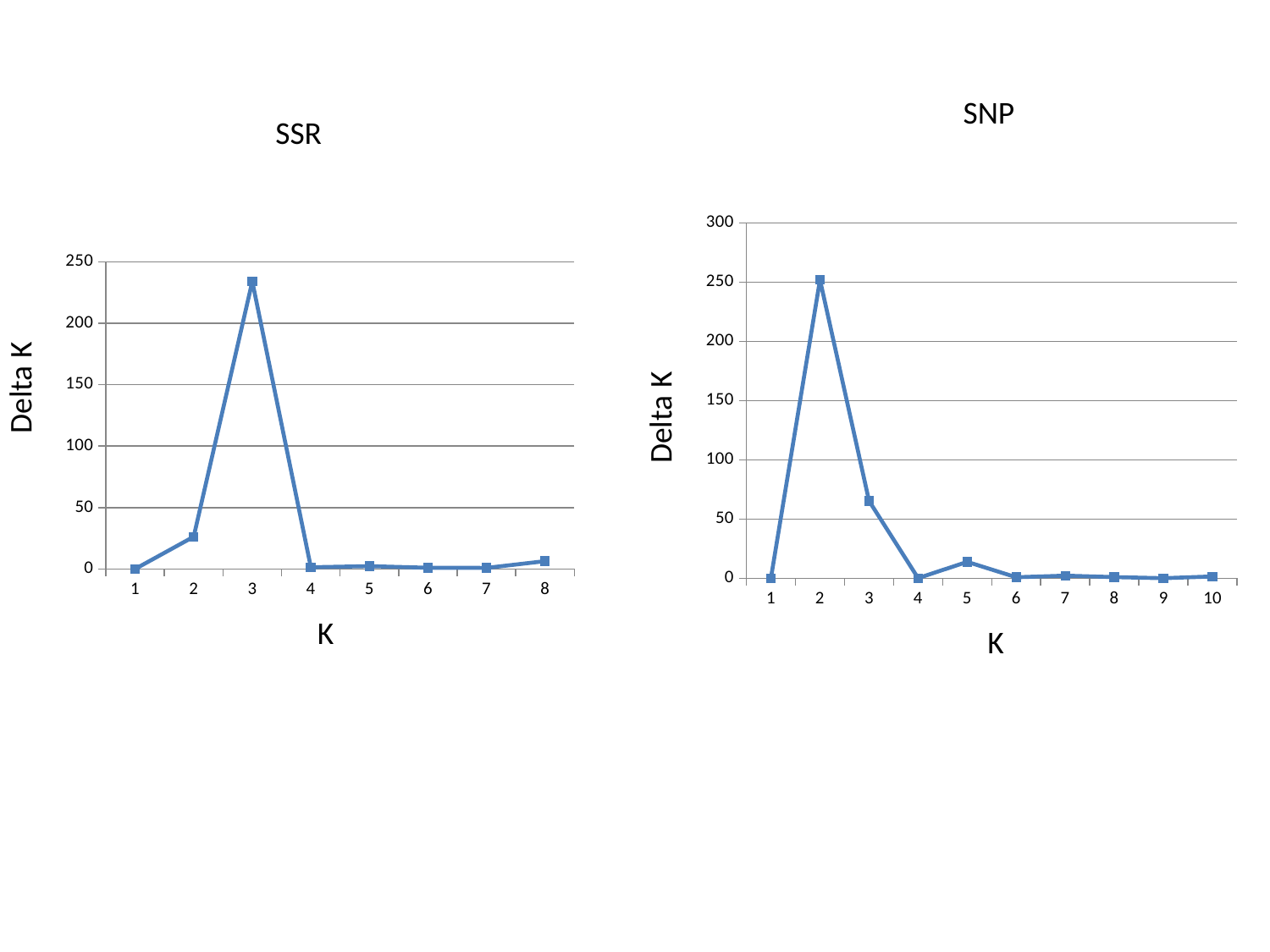

SNP
 SSR
### Chart
| Category | |
|---|---|
### Chart
| Category | |
|---|---|
| 1 | 0.0 |
| 2 | 26.21177454976936 |
| 3 | 234.0637302156871 |
| 4 | 1.34446263285041 |
| 5 | 2.2625767061384 |
| 6 | 0.9510280065918815 |
| 7 | 0.8381922145172895 |
| 8 | 6.3336122748562795 |Delta K
Delta K
K
K

Supplement: Additional file 5 — Evanno test for the structure analysis. [file 1471-2156-13-48-S5.pptx]

## Slide 1
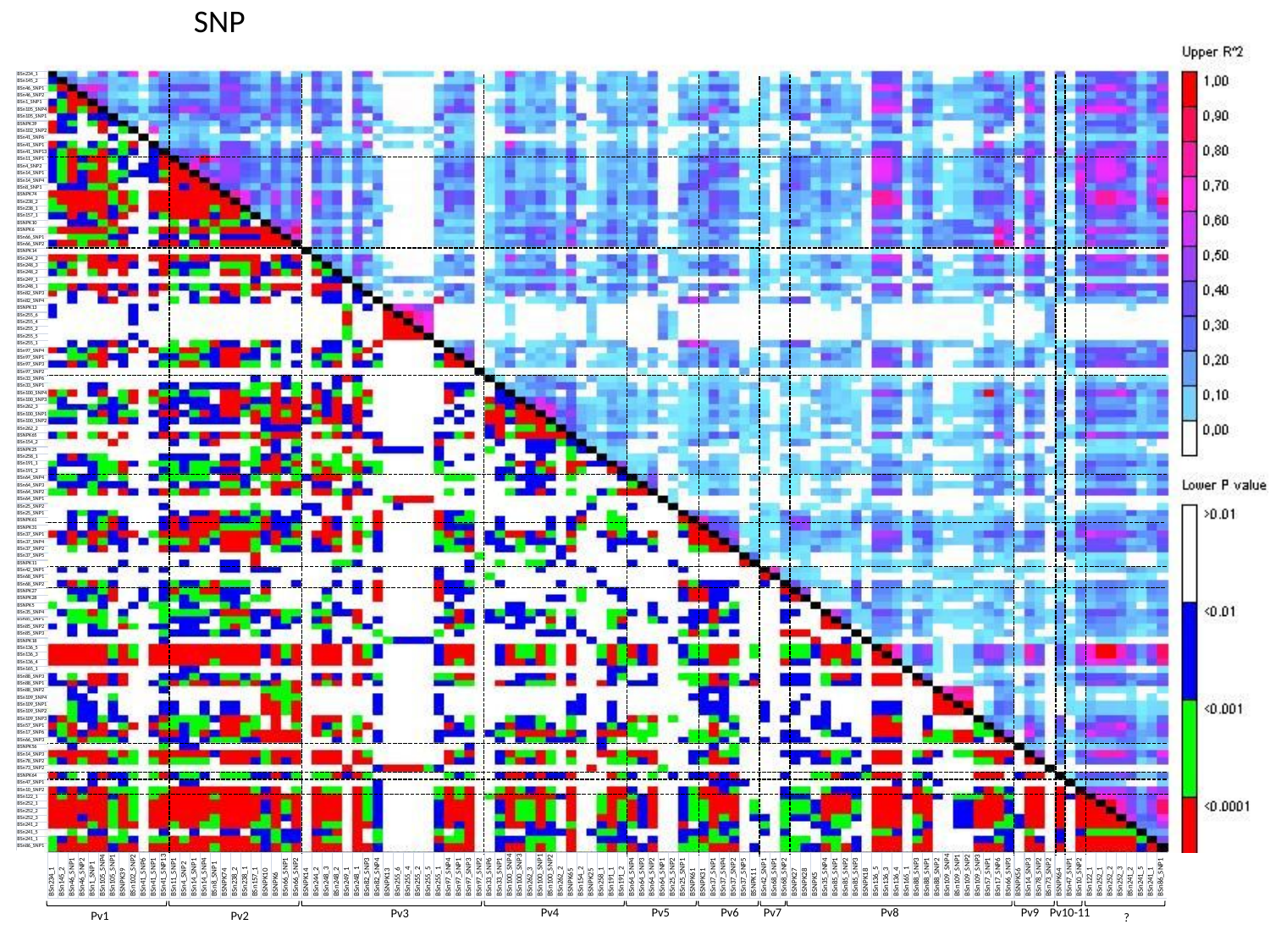

SNP
Pv4
Pv5
Pv6
Pv7
Pv8
Pv9
Pv10-11
Pv3
Pv2
Pv1
?

## Slide 2
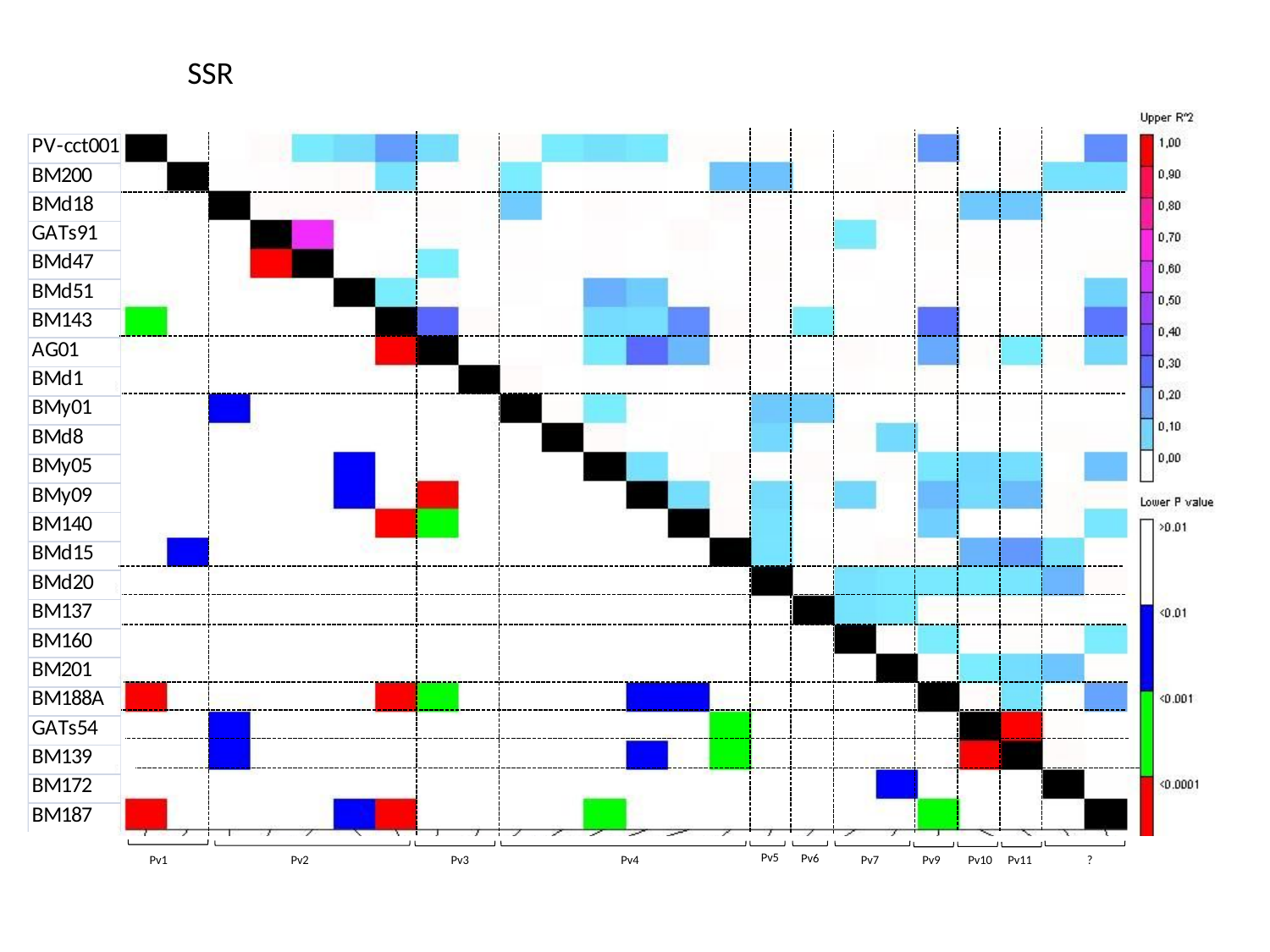

SSR
Pv5
Pv6
Pv1
Pv2
Pv3
Pv4
Pv7
Pv9
Pv10
Pv11
?

Supplement: Additional file 6 — Linkage disequilibrium heat maps(r2vs.p-value). [file 1471-2156-13-48-S6.pptx]
