# Supplementary material for: Gene-Based Single Nucleotide Polymorphism Markers for Genetic and Association Mapping in Common Bean
Source: BMC Genet. 2012 Jun 26;13:48. doi: 10.1186/1471-2156-13-48 (PMC3464600; doi:10.1186/1471-2156-13-48)
Supplement: Additional file 4 — List of the diversity panel genotypes. [file 1471-2156-13-48-S4.docx]

Supplementary Table 4. Common bean genotypes used in this study. Principal characteristics in terms of gene pool, growth habit, seed size, and phaseolin allele are presented.

| **Genotype** | **Phaseolin** ^a^ | **Seed size** ^b^ | **Growth habit** ^c^ | **Race** ^d^ | **Gene pool** |
| --- | --- | --- | --- | --- | --- |
| G11957 | T | L | III | NG1 | Andean |
| G11982 | T | L | II | NG1 | Andean |
| G13094 | T | L | I | NG1 | Andean |
| G16115 | T | L | I | NG1 | Andean |
| G1688 | T | M | II | NG1 | Andean |
| G17070 | T | L | I | NG1 | Andean |
| G17076 | T | L | I | NG1 | Andean |
| G18255 | T | L | I | NG1 | Andean |
| G1836 | T | M | II | NG1 | Andean |
| G18942 | T | L | I | NG1 | Andean |
| G1939 | T | M | II | NG1 | Andean |
| G21210 | T | L | I | NG1 | Andean |
| G22247 | T | L | II | NG1 | Andean |
| G2875 | T | L | II | NG1 | Andean |
| G3157 | T | L | I | NG1 | Andean |
| G4001 | T | L | II | NG1 | Andean |
| G4534 | CA | L | I | NG1 | Andean |
| G4906 | CA | L | I | NG1 | Andean |
| G5142 | T | L | II | NG1 | Andean |
| G5273 | T | L | II | NG1 | Andean |
| G5625 | T | L | I | NG1 | Andean |
| G6639 | TM | L | I | NG1 | Andean |
| G738 | T | L | I | NG1 | Andean |
| G7776 | T | M | II | NG1 | Andean |
| G7945 | TM | L | I | NG1 | Andean |
| G9846 | T | L | I | NG1 | Andean |
| G12689 | T | L | I | NG1 | Andean |
| AND1005 | T | L | II | NG2 | Andean |
| G11512 | T | M | I | NG2 | Andean |
| G11564 | T | L | IIA | NG2 | Andean |
| G11585 | T | L | I | NG2 | Andean |
| G11727 | T | L | III | NG2 | Andean |
| G11759A | T | M | IIA | NG2 | Andean |
| G12517 | T | M | III | NG2 | Andean |
| G13595 | T | L | III | NG2 | Andean |
| G13910 | T | M | III | NG2 | Andean |
| G13911 | T | M | III | NG2 | Andean |
| G14253 | T | L | I | NG2 | Andean |
| G16104E | T | M | I | NG2 | Andean |
| G16110A | T | L | II | NG2 | Andean |
| G16346 | T | L | IIB | NG2 | Andean |
| G1678 | T | L | II | NG2 | Andean |
| G17168 | T | L | IIN | NG2 | Andean |
| G18264 | T | L | III | NG2 | Andean |
| G23829 | T | M | II | NG2 | Andean |
| G2563 | T | L | I | NG2 | Andean |
| G4644 | T | L | I | NG2 | Andean |
| G4672 | CA | M | III | NG2 | Andean |
| G5034 | T | L | I | NG2 | Andean |
| G5170 | T | L | I | NG2 | Andean |
| G5708 | T | L | I | NG2 | Andean |
| G5849 | H | L | III | NG2 | Andean |
| G6873 | T | L | I | NG2 | Andean |
| G7895 | T | L | I | NG2 | Andean |
| G9335 | T | M | III | NG2 | Andean |
| G9603 | T | L | III | NG2 | Andean |
| G9855 | T | L | IIB | NG2 | Andean |
| PVA1111 | T | L | I | NG2 | Andean |
| G19497 | PA | M | IIIB | NG2 | Andean |
| G23484G | T | S | III | NG2 | Andean |
| DRK47 | T | L | I | P1 | Andean |
| G11521 | C | L | I | P1 | Andean |
| G12529 | T | S | III | P1 | Andean |
| G14016 | T | M | II | P1 | Andean |
| G19833 | H | M | III | P1 | Andean |
| G19842 | T | L | II | P1 | Andean |
| G19860 | T | M | III | P1 | Andean |
| G22147 | T | L | I | P1 | Andean |
| G23604 | T | M | II | P1 | Andean |
| G2567 | T | L | II | P1 | Andean |
| G2686 | T | L | I | P1 | Andean |
| G4494 | T | L | I | P1 | Andean |
| G4547 | H | L | I | P1 | Andean |
| G4721 | H | L | II | P1 | Andean |
| G4739 | T | M | II | P1 | Andean |
| G8209 | C | L | IIIB | P1 | Andean |
| PVA773 | T | L | I | P1 | Andean |
| G21720 | T | L | I | P1 | Andean |
| G14659 | T | L | II | P1 | Andean |
| G19839 | na | na | na | na | Andean |
| G2333 | S | M | IV | D2 | Mesoamerican |
| DOR364 | S | M | II | M1 | Mesoamerican |
| BAT477 | na | na | na | na | Mesoamerican |
| G3513 | na | na | na | na | Mesoamerican |
| BAT881 | na | na | na | na | Mesoamerican |
| G21212 | na | na | na | na | Mesoamerican |
| AFR619 | na | na | na | na | na |
| CAL143 | na | na | na | na | na |
| CAL96 | na | na | na | na | na |
| SAB258 | na | na | na | na | na |
| SAB645 | na | na | na | na | na |
| SEQ1003 | na | na | na | na | na |
| SEQ1027 | na | na | na | na | na |

a. Phaseolin allele as determined by CIAT Genetic Resource

b. Seed size defined as: small (S) = below 25 g/100 seeds, medium (M) = between 25 and 40 g/100 seeds, large (L) = above 40 g/100 seeds, as classified by Hidalgo *et al.* (1992)

c. Growth habit defined as I = determinate bush, II = indeterminate bush, III = indeterminate prostrate, IV = indeterminate climbing, as classified by Hidalgo *et al.* (1992)

d. Races: D Durango G Guatemala, NG1 Nueva Granada group1, NG2 Nueva Granda group 2, P Peru, M Mesoamerica.
